# Multimodality Imaging Approach in the Diagnosis of Constrictive Pericarditis

**DOI:** 10.3390/diagnostics16050707

**Published:** 2026-02-27

**Authors:** Lucia La Mura, Francesca Musella, Marianna D’Amato, Maria Lembo, Costantino Mancusi, Marco Ferrone, Ilaria Fucile, Ciro Santoro, Raffaele Izzo, Giovanni Esposito

**Affiliations:** 1 Department of Advanced Biomedical Sciences, “Federico II” University of Naples, 80131 Naples, Italy; maria.lembo@unina.it (M.L.); costantino.mancusi@unina.it (C.M.); ilaria.fucile@unina.it (I.F.); raffaele.izzo@unina.it (R.I.); giovanni.esposito2@unina.it (G.E.); 2 Department of Clinical Science and Education, Södersjukhuset, Karolinska Institutet, 118 83 Stockholm, Sweden; francmusella@gmail.com; 3 Cardiology Department, Santa Maria delle Grazie Hospital, 80078 Pozzuoli, Italy; 4 Servicio de Cardiologia, Hospital Central de la Defensa Gomez Ulla, 28047 Madrid, Spain; marianna.damato89@gmail.com; 5 Catheterization Laboratory, Division of Cardiology, Montevergine Clinic, 83013 Mercogliano, Italy; marco.ferrone1@gmail.com; 6 Department of Pharmacy, Health and Nutritional Sciences, University of Calabria, Rende (CS), 87036 Arcavacata, Italy; ciro.santoro@unical.it

**Keywords:** multimodality imaging, constrictive pericarditis, cardiac magnetic resonance, cardiac CT, CMR, PET imaging, nuclear imaging, echocardiography, cardiac inflammatory diseases

## Abstract

Constrictive pericarditis (CP) results from persistent or insufficiently controlled pericardial inflammation arising from diverse etiologies. It remains a challenging clinical condition, typically presenting with non-specific symptoms that demand a high degree of clinical suspicion and meticulous imaging assessment. As CP progressively impairs both left- and right-sided cardiac function, it can lead to overt heart failure and a marked decline in quality of life, making early recognition crucial. Multimodality imaging plays an essential role in the diagnostic and prognostic evaluation of CP, enabling distinction from restrictive cardiomyopathy (RCM), detection of active pericardial inflammation, and guidance of therapeutic decision-making. Echocardiography provides key hemodynamic insights, including ventricular interdependence and respiratory variation in flow patterns. Cardiac Computed Tomography (CT) offers high-resolution delineation of pericardial thickening and calcification, while Cardiac magnetic resonance (CMR) allows comprehensive characterization of pericardial anatomy, myocardial–pericardial coupling, and inflammatory burden through late gadolinium enhancement (LGE) and parametric mapping. Additionally, positron emission tomography (PET) imaging can identify metabolically active inflammation, aiding in determining the suitability of medical therapy versus pericardiectomy. By integrating these complementary modalities, clinicians can achieve greater diagnostic precision, refine risk stratification, and tailor management strategies, ultimately improving outcomes for patients with constrictive pericarditis.

## 1. Introduction

This narrative review was conducted using a structured search of PubMed, Scopus, and Web of Science from 1990 to 2025. Search terms included combinations of “constrictive pericarditis”, “multimodality imaging”, “echocardiography”, “cardiac magnetic resonance”, “computed tomography”, “positron emission tomography”, and “hemodynamics”. International society guidelines and position statements were preferentially included. Given heterogeneity in patient populations, etiologies, and study eras, emphasis was placed on consistent physiological principles and reproducible diagnostic findings across modalities rather than isolated diagnostic accuracy estimates.

### 1.1. Normal Pericardial Anatomy and Physiology

The pericardium consists of a visceral serous layer adherent to the epicardium and a fibrous parietal layer forming a relatively non-distensible sac containing 15–50 mL of fluid. Under normal conditions, the pericardium limits acute cardiac dilation, maintains ventricular coupling, and transmits intrathoracic pressure changes uniformly to all cardiac chambers. Because the pericardium is compliant at low volumes but rapidly becomes stiff at higher volumes, it plays a key role in ventricular interdependence. During inspiration, decreased intrathoracic pressure is transmitted equally to all chambers, allowing balanced filling of both ventricles without significant septal shift.

### 1.2. Pathophysiology

CP is a complex clinical syndrome characterized by progressive loss of pericardial elasticity due to inflammation, fibrosis, and, in advanced stages, calcification, ultimately resulting in impaired cardiac pump performance [1]. Under this condition, the pericardium becomes thickened, rigid, and adherent to the epicardial surface, effectively encasing the heart and limiting its normal interaction with intrathoracic pressures. This pathological constraint restricts ventricular diastolic filling and leads to dissociation between intrathoracic and intracardiac pressures, accompanied by exaggerated ventricular interdependence. These mechanisms culminate in diastolic dysfunction, elevation and equalization of venous and ventricular diastolic pressures, respiratory variation in ventricular filling, and ultimately reduced cardiac output [2].

### 1.3. Etiology

The etiological spectrum of CP has evolved over time and varies significantly by geographic region. In contemporary clinical practice in developed countries, idiopathic disease represents the most common cause, followed by postsurgical and radiation-induced pericardial injury. In contrast, tuberculosis remains the leading cause of CP in developing regions [3]. Importantly, the risk of progression from acute pericarditis to constrictive physiology is strongly dependent on the underlying etiology. The highest incidence is observed in purulent pericarditis (52.74 cases per 1000 person-years) and tuberculous pericarditis (31.65 cases per 1000 person-years), whereas the risk is substantially lower—approximately an order of magnitude—for neoplastic pericarditis, systemic rheumatic disease, and post–cardiac injury syndromes. When idiopathic or viral acute pericarditis is appropriately treated, the development of CP is rare, occurring in only 0.76 cases per 1000 person-years [4].

### 1.4. Clinical Findings

Clinically, CP often presents with nonspecific symptoms that may delay diagnosis. Dyspnea, fatigue, orthopnea, and peripheral edema are common presenting complaints, and the classic clinical picture is frequently dominated by isolated right-sided heart failure. Venous congestion, hepatomegaly, pleural effusions, and ascites are typical manifestations in advanced disease. Kussmaul’s sign, originally described in CP, reflects impaired right ventricular filling and elevated right atrial pressure, although it is not specific and may be observed in other conditions associated with increased right atrial pressure [5].

Laboratory evaluation in suspected CP should include assessment of systemic inflammation through C-reactive protein (CRP) and erythrocyte sedimentation rate (ESR), as well as cardiac biomarkers such as B-type natriuretic peptide and troponin. While these markers may provide supportive information regarding inflammatory activity or myocardial involvement, they lack specificity for CP. Electrocardiographic findings are similarly nonspecific and may include low-voltage QRS complexes, atrial fibrillation, and P-wave abnormalities suggestive of atrial enlargement, reflecting chronic atrial pressure overload [1].

### 1.5. Clinical Spectrum and Therapeutic Strategies of CP

Constrictive pericarditis encompasses a spectrum of disease states, and therapeutic strategies are determined largely by disease chronicity and the potential for reversibility. Subacute CP represents an early stage characterized by active inflammation and partial preservation of pericardial compliance. Transient CP is a recognized variant of subacute disease that resolves spontaneously or with medical therapy within 3 to 6 months and is associated with a more favorable prognosis compared with chronic CP. Effusive-constrictive pericarditis (ECP) is defined by persistently elevated right atrial pressure despite normalization of intrapericardial pressure following pericardial effusion drainage. In contrast, chronic CP is typically irreversible due to established fibrotic remodeling of the pericardium and generally requires radical pericardiectomy as definitive therapy [5].

Management of CP is guided by the identification and treatment of the underlying etiology, particularly in potentially reversible forms. In transient or subacute CP, anti-inflammatory therapy is pursued before consideration of surgical intervention [6]. Nonsteroidal anti-inflammatory drugs and colchicine are most commonly used as first-line therapy, with corticosteroids reserved for patients with contraindications to NSAIDs, such as chronic kidney disease, increased bleeding risk, or active peptic ulcer disease. Treatment regimens generally mirror those used for acute pericarditis; however, therapy duration is often longer, with treatment extending for 3 to 6 months followed by gradual tapering. In tuberculous constrictive pericarditis, antituberculous therapy may lead to resolution of constrictive physiology, and adjunctive corticosteroids may further enhance clinical improvement [7].

In ECP, management parallels that of transient CP, with pericardiocentesis as the initial therapeutic step to relieve pericardial effusion. In cases refractory to aggressive anti-inflammatory therapy, pericardiectomy with extensive epicardiectomy may be required due to involvement of the visceral pericardium [8].

## 2. Echocardiography

Echocardiography represents the first-line imaging modality in the diagnostic evaluation of CP. Owing to its widespread availability, real-time assessment of cardiac hemodynamics, and ability to detect characteristic features of constrictive physiology, echocardiography plays a central role in the initial identification of CP and in guiding subsequent multimodality imaging.

**Figure 1:**
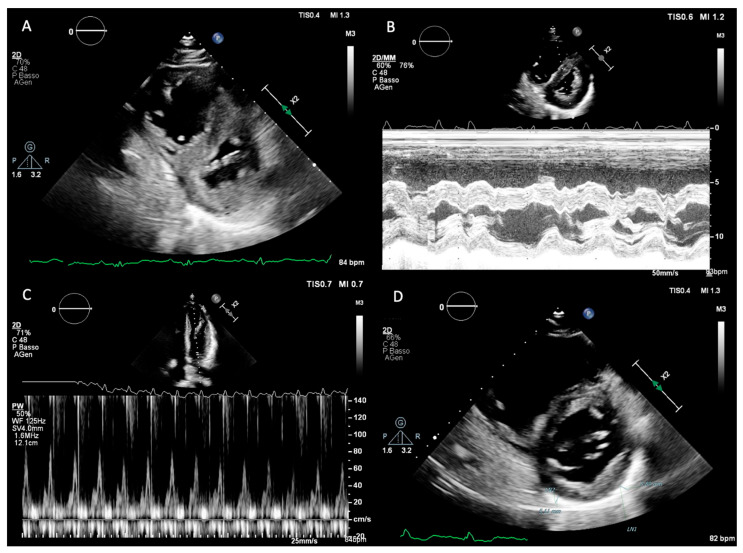
Ecocardiography findings in CP: respirophasic interventricular septal motion observed on both 2D echocardiography (**A**) and M-mode (**B**); respiratory variation in transmitral inflow (**C**); Measurement of pericardial thickness by two-dimensional echocardiography (**D**).

Normal pericardial thickness is 2 mm or less, and a rigid and/or thickened pericardium constitutes the anatomic substrate responsible for constrictive physiology. Despite isolated reports suggesting the utility of M-mode and two-dimensional (2D) echocardiography in detecting pericardial thickening (Figure 1D), the reliability of transthoracic echocardiography for this purpose remains limited due to technical factors such as transducer position, gain and greyscale settings, and reverberation artifacts. As a result, echocardiography is primarily used to assess the functional consequences of pericardial disease rather than to provide a direct anatomical evaluation of the pericardium.

Left ventricular (LV) systolic function, as assessed by LV ejection fraction, is typically preserved in CP. One of the hallmark echocardiographic features is respirophasic interventricular septal motion, which can be observed on both M-mode and 2D echocardiography (Figure 1A,B). This phenomenon is induced by abrupt changes in ventricular volumes and reflects enhanced ventricular interdependence. During early inspiration, reduced LV filling leads to a sudden leftward shift in the interventricular septum, whereas during expiration, improved LV filling allows the septum to return toward its normal position. This hemodynamic abnormality results in inspiratory septal shift to the left, a plethoric inferior vena cava, and late-diastolic expiratory flow reversal in the hepatic veins [9,10].

Doppler echocardiography provides further insights into diastolic filling dynamics. Because LV diastolic pressure is elevated and virtually all LV filling occurs in early diastole, transmitral inflow typically demonstrates an increased E-wave velocity with a shortened deceleration time, usually less than 160 ms, and a small or absent A-wave. With inspiration, dissociation between intrathoracic and intracardiac pressures results in a decrease in the initial driving pressure for LV filling. Consequently, peak mitral E-wave velocity decreases by more than 25% during the first beat of inspiration, accompanied by prolongation of the isovolumic relaxation time, usually exceeding 20% (Figure 1C). Ventricular interdependence produces reciprocal changes in right-sided filling, with an inspiratory increase in peak tricuspid E-wave velocity greater than 40%. Reverse changes occur during expiration in both ventricles [11].

Advanced echocardiographic techniques, including tissue Doppler imaging and myocardial deformation analysis, further refine the assessment of CP. Baseline 2D echocardiography often demonstrates a hyperdynamic mitral annulus with exaggerated longitudinal motion in patients with CP (12). Unless coexisting cardiomyopathy is present, medial mitral annular early diastolic velocity (e′) is often exaggerated, typically measuring ≥9 cm/s. Pericardial adhesions tether the lateral mitral annulus while sparing the interventricular septum, giving rise to the phenomenon of annulus reversus, in which the usually lower septal e′ velocity exceeds the lateral mitral annular e′ velocity. A similar concept applies to myocardial deformation imaging, described as strain reversus, defined by a lateral LV longitudinal strain–to–septal wall longitudinal strain ratio < 0.96 [10,11,12,13].

For the same pathophysiological reasons, marked epicardial dysfunction in CP leads to impairment of circumferential shortening and twist mechanics, whereas subendocardial myocardial deformation, reflected by longitudinal strain, remains relatively preserved. In contrast, longitudinal strain is significantly reduced in RCM, predominantly affecting subendocardial fibers oriented in a longitudinal direction [14]. These distinct patterns of longitudinal and circumferential LV mechanics are readily assessed by 2D speckle-tracking echocardiography, a relatively angle-independent technique that tracks unique intramyocardial features within greyscale B-mode images.

Differentiation between CP and RCM remains a complex and often challenging process. Because CP represents a potentially curable cause of heart failure, whereas therapeutic options for RCM are limited, an accurate distinction between these two entities is of critical clinical importance. Although CP and RCM differ with respect to etiology, prognosis, and treatment, they share a common clinical presentation characterized by predominantly right-sided heart failure in the absence of significant LV systolic dysfunction or primary valvular disease, due to impaired ventricular diastolic filling.

On 2D echocardiography, increased pericardial thickness is characteristic of CP, although systemic venous congestion is present in both CP and RCM. A plethoric inferior vena cava and engorged hepatic veins are expected findings in both conditions. In primary (idiopathic) RCM, ventricular cavity size and wall thickness tend to be normal, while severe atrial enlargement is often present [15]. Although hemodynamics are similar in primary and secondary (infiltrative) forms of RCM, ventricular wall thickness is commonly increased in infiltrative diseases [16].

Doppler echocardiography and myocardial deformation imaging provide the most discriminative parameters for differentiating CP from RCM. In both conditions, mitral and tricuspid Doppler inflow patterns are characterized by predominant early diastolic velocities (E-wave) with shortened deceleration time, reflecting rapid early ventricular filling. A critical distinguishing feature is the presence of respiratory flow variation in CP, which is absent in RCM. Hepatic vein Doppler interrogation in CP typically demonstrates decreased expiratory diastolic forward velocities with prominent expiratory diastolic flow reversals.

Among all echocardiographic parameters, mitral annular tissue Doppler assessment is perhaps the most useful for distinguishing CP from RCM. As myocardial stiffening progresses and relaxation becomes delayed, e′ velocities are reduced, a hallmark feature of RCM [17]. In CP, elevated filling pressures are not caused by intrinsic myocardial dysfunction or impaired relaxation but by extrinsic pericardial constraint. Lateral cardiac motion is limited, and ventricular filling relies predominantly on longitudinal motion. Consequently, mitral annular e′ velocities are normal or paradoxically increased despite elevated filling pressures, a phenomenon termed annulus paradoxus [18]. In addition, tethering of the LV free wall may result in reversal of the normal relationship between medial and lateral mitral annular tissue Doppler velocities, with medial e′ typically exceeding lateral e′ and often measuring >7 cm/s, a finding referred to as annulus reversus.

Finally, myocardial deformation imaging may assume an increasingly important role in the differentiation of CP and RCM. Preliminary data suggest that patients with CP exhibit markedly abnormal circumferential deformation, torsion, and untwisting velocity with relative sparing of longitudinal mechanics, whereas RCM is associated with abnormal longitudinal mechanics, most pronounced at the basal segments, and relative preservation of LV rotational mechanics [14].

## 3. Cardiac Magnetic Resonance

CMR has become a cornerstone in the diagnostic evaluation of CP, owing to its unparalleled ability to integrate high-resolution anatomical imaging with advanced tissue characterization and dynamic hemodynamic assessment. While transthoracic echocardiography remains the initial modality for identifying constrictive physiology, CMR provides a uniquely comprehensive assessment of the pericardium and adjacent myocardium at structural, functional, and biological levels. Consequently, contemporary recommendations position CMR at the forefront of the diagnostic pathway, particularly when echocardiographic findings are equivocal or when differentiation between CP and RCM is clinically necessary [19,20].

The diagnostic strength of CMR lies in its integration of complementary imaging sequences—most notably T2-weighted imaging and LGE—that together characterize inflammation, edema, hypervascularity, and fibrosis. Increasingly, CMR is not merely confirmatory but serves as a powerful phenotyping tool, enabling clinicians to differentiate reversible, inflammation-driven constriction from chronic fibrotic disease that is unlikely to respond to medical therapy and instead requires surgical intervention.

**Figure 2:**
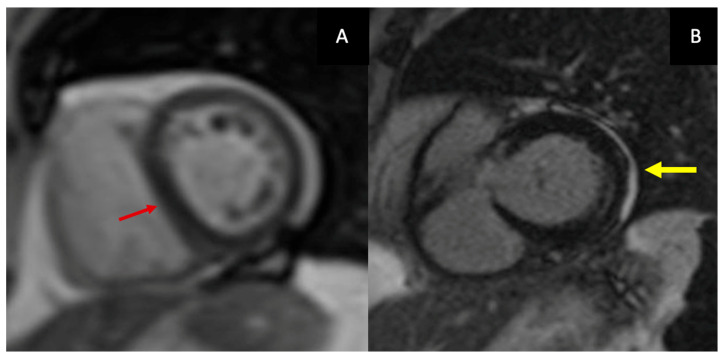
CMR findings in CP: real-time free-breathing cine imaging shows respiratory-induced septal shift (**A**) (red arrow); pericardial LGE (**B**) (yellow arrow).

Anatomical assessment begins with black-blood spin-echo sequences, which allow evaluation of pericardial anatomy and thickness, and cine steady-state free-precession (SSFP) sequences, which provide detailed visualization of pericardial morphology and ventricular interaction. Cine imaging depicts classical features of CP, including tubular or conical ventricular geometry, impaired diastolic expansion, exaggerated ventricular interdependence, and the early-diastolic septal “bounce”. Although pericardial thickening >4 mm is traditionally considered suggestive of CP, thickness alone is an unreliable discriminator: many patients with established constriction, particularly those with early inflammatory disease or postsurgical adhesions, exhibit normal pericardial thickness [21]. Current recommendations, therefore, emphasize the integration of anatomic, functional, and tissue-based parameters rather than relying on thickness in isolation. Furthermore, CMR allows direct visualization of constrictive physiology through real-time free-breathing cine imaging, which captures exaggerated ventricular interdependence and respiratory-induced septal shift (Figure 2A).

The ability of CMR to detect and quantify pericardial inflammation represents one of its most transformative contributions to CP evaluation. T2-weighted short-tau inversion recovery (STIR) imaging identifies pericardial edema as areas of high signal intensity, reflecting water-rich inflamed tissue. The presence of edema carries major therapeutic implications, indicating active inflammation and supporting the use of targeted anti-inflammatory therapy—including NSAIDs, colchicine, corticosteroids, and interleukin-1 inhibitors—while reducing the risk of premature surgical referral. The ESC Guidelines highlight the fundamental importance of edema detection for accurately identifying reversible disease and guiding treatment escalation or de-escalation [20].

LGE provides complementary information on inflammatory activity and fibrosis and is central to defining the chronicity of disease (Figure 2B).

In clinical practice, LGE is assessed according to both the thickness and circumferential extent of pericardial enhancement across basal, mid-ventricular, and apical short-axis levels. Based on these parameters, enhancement patterns can be interpreted along a spectrum from none to severe. Mild enhancement reflects limited involvement—either thin but circumferential (>50%) or focal (<50%) but slightly thickened—whereas moderate enhancement indicates ≥50% circumferential extent for <3 slices and with thickness ≤ 3 mm or <50% circumferential extent involving ≥3 slices and with thickness > 3 mm. Severe enhancement is characterized by marked thickening (>3 mm) with extensive circumferential involvement at several levels. This semi-quantitative approach to pericardial LGE is particularly valuable in patients’ follow-up and in guiding evidence-based therapeutic decisions [19].

The distribution of LGE is equally important in distinguishing CP from RCM. Enhancement confined to the pericardium supports CP, whereas characteristic myocardial enhancement patterns—such as subendocardial deposition in amyloidosis or mid-wall distribution in myocarditis or sarcoidosis—indicate alternative diagnoses. Pericardial LGE should, however, be interpreted with caution: its presence frequently reflects ongoing inflammation, hypervascularity, and neovascular remodeling rather than fixed fibrosis. LGE is typically the last imaging biomarker to normalize; therefore, resolution or near-resolution of pericardial enhancement provides the most definitive imaging evidence of inflammatory resolution [22]. Conversely, chronic or unresolved inflammation may evolve into dense fibrinous adhesions and eventually calcific transformation of the pericardium.

Clinically, the comprehensive information obtained from CMR has direct therapeutic implications. Patients demonstrating edema and mild-to-severe LGE patterns generally exhibit an inflammatory phenotype with a high likelihood of reversibility under intensive anti-inflammatory therapy. By contrast, patients without LGE typically show predominant fibrosis or calcification, characterized by a structurally rigid pericardium, and are most appropriately referred for early pericardiectomy.

From a clinical management perspective, a high likelihood of reversibility is best defined by integrating the extent, distribution, and temporal evolution of inflammatory findings rather than by a single imaging threshold. Extensive pericardial edema on T2-weighted sequences together with moderate-to-severe circumferential LGE—particularly when involving multiple ventricular levels—has been most consistently associated with active inflammatory constriction and favorable response to anti-inflammatory therapy [6,19]. Conversely, minimal or absent enhancement in the setting of marked pericardial thickening or calcification strongly suggests a chronic fibrotic “burnt-out” phenotype unlikely to improve with medical treatment. Serial imaging further refines prognostic assessment: reduction in T2 signal intensity and progressive decrease in LGE burden correlate with clinical improvement and resolution of constrictive physiology, whereas persistent or increasing enhancement may indicate ongoing inflammation or evolution toward irreversible fibrosis [22].

Follow-up CMR is commonly performed after approximately 2–3 months of optimized anti-inflammatory therapy, although timing should be individualized according to symptom severity and clinical trajectory [19,20]. Echocardiography may serve as an interim tool to monitor hemodynamic improvement, while repeat CMR provides definitive reassessment of inflammatory activity and structural remodeling. Imaging findings should be interpreted in conjunction with systemic inflammatory biomarkers, particularly C-reactive protein and erythrocyte sedimentation rate, which—when elevated and subsequently declining with therapy—support an active inflammatory phenotype and treatment responsiveness [6,20]. This integrated imaging–clinical approach enables more precise identification of patients who may benefit from continued medical therapy versus those who should be considered for timely surgical pericardiectomy.

## 4. Cardiac Computed Tomography

Cardiac CT plays a pivotal and complementary role in the multimodality imaging evaluation of CP, primarily owing to its excellent spatial resolution and its ability to provide comprehensive anatomical assessment of the pericardium and surrounding thoracic structures. Although CT has traditionally been considered a second-line modality, contemporary guidelines and expert consensus documents now recognize its central role in selected clinical scenarios, particularly when pericardial calcification is suspected, when CMR is contraindicated or inconclusive, or when detailed preoperative planning is required [19,20,23].

**Figure 3:**
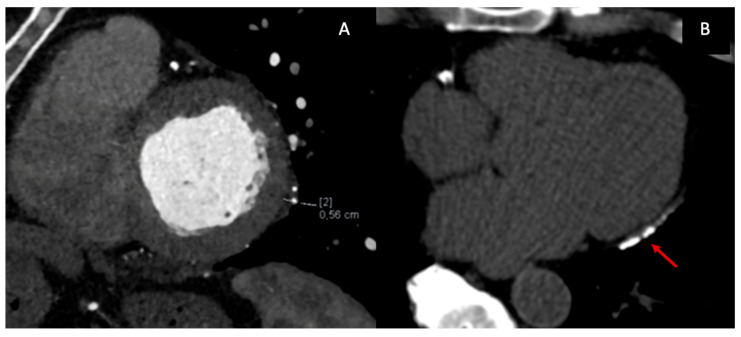
Cardiac CT findings in CP: ECG-gated contrast-enhanced CT shows focal pericardial thickening (**A**); ECG-gated no contrast-enhanced CT can detect the presence and extension of pericardial calcifications (**B**) (red arrow).

From an anatomical perspective, CT represents the reference standard for evaluating pericardial thickness, morphology, and distribution. ECG-gated contrast-enhanced CT enables precise delineation of the pericardial layers, which normally measure less than 2 mm, and allows detection of focal or circumferential thickening, asymmetric involvement, and pericardial adhesions (Figure 3A). While a thickness > 4 mm is classically considered suggestive of constriction, CT—similar to CMR—has demonstrated that a substantial proportion of patients with surgically confirmed CP may exhibit normal or only mildly increased pericardial thickness, underscoring the limited specificity of thickness alone as a diagnostic criterion [24,25]. Consequently, CT findings must be interpreted in conjunction with functional and hemodynamic data.

One of the most distinctive contributions of CT to the diagnosis of CP is the detection and characterization of pericardial calcifications (Figure 3B). CT is markedly superior to echocardiography and CMR for identifying and mapping calcific deposits, which may be focal or diffuse and often involve the diaphragmatic surface, atrioventricular grooves, and basal ventricular segments. The presence of extensive calcification strongly supports a chronic, non-inflammatory phenotype of CP and has important therapeutic implications, as these patients are unlikely to respond to anti-inflammatory therapy and are typically referred for pericardiectomy [23,26]. Furthermore, CT allows accurate assessment of the extent, thickness, and distribution of calcifications, information that is crucial for surgical risk stratification and operative planning [24].

Beyond structural assessment, contrast-enhanced CT increasingly contributes to the evaluation of pericardial inflammation. Delayed post-contrast imaging can demonstrate pericardial enhancement reflecting hyperemia and increased vascular permeability, suggesting ongoing inflammatory activity. More recently, dual-energy CT has enabled iodine-based material decomposition and quantitative iodine mapping of the pericardium, providing a surrogate marker of inflammatory burden analogous to LGE on CMR [19,27]. Photon-counting detector CT represents a further technological advance, offering improved contrast-to-noise ratio, higher spatial resolution, and enhanced tissue characterization while potentially reducing radiation exposure, although its role in routine CP assessment is still evolving [27].

In addition to anatomical and inflammatory characterization, CT can provide functional information relevant to constrictive physiology. ECG-gated cine CT acquisitions allow dynamic assessment of cardiac motion throughout the cardiac cycle, enabling visualization of impaired diastolic expansion, abnormal ventricular coupling, and septal flattening. Although temporal resolution remains inferior to echocardiography and CMR, cine CT may be particularly useful in patients with poor acoustic windows or contraindications to MRI. Emerging applications of CT-derived myocardial strain have shown potential in differentiating CP from RCM by demonstrating preserved or relatively increased longitudinal strain with impaired circumferential mechanics; however, these techniques remain largely investigational and are not yet incorporated into standard diagnostic algorithms [24,26].

CT plays a particularly important role in preoperative planning for pericardiectomy. Surgical outcomes in CP are influenced by the extent and distribution of pericardial disease, the presence of dense calcifications, and involvement of adjacent structures such as the phrenic nerves, coronary arteries, and great vessels. CT provides a detailed anatomical roadmap, enabling identification of regions with dense calcification or myocardial adherence and allowing surgeons to anticipate technical challenges, optimize the surgical approach, and minimize perioperative complications [19,23].

Finally, the large field of view of CT allows comprehensive evaluation of extracardiac findings, which is essential for etiological assessment and differential diagnosis. CT can identify pulmonary, pleural, or mediastinal abnormalities suggestive of tuberculosis, malignancy, prior radiation therapy, or systemic inflammatory disease—conditions frequently associated with CP. Detection of lymphadenopathy, pleural thickening, lung parenchymal disease, or mediastinal masses may prompt additional investigations and significantly influence clinical management [20,25]. Moreover, CT enables exclusion of alternative causes of right-sided heart failure, such as chronic thromboembolic pulmonary hypertension or intrinsic lung disease. In addition, CT is often preferred over CMR in patients with contraindications to MRI—such as non-MRI-compatible implanted devices, severe claustrophobia, or inability to tolerate prolonged breath-holding—and when precise characterization and mapping of pericardial calcification are required for surgical planning.

## 5. Nuclear Imaging

Nuclear imaging has emerged as a powerful adjunct in the multimodality assessment of CP, primarily by enabling direct visualization and quantification of metabolically active pericardial inflammation. Unlike echocardiography, cardiac CT, and CMR, which predominantly assess structural and functional consequences of pericardial disease, PET imaging provides unique biological information by detecting glucose or fibroblast-associated tracer uptake, thereby identifying active inflammatory or remodeling processes within the pericardium. This capability is particularly valuable in differentiating active, potentially reversible constriction from chronic, fibrotic disease and in guiding imaging-based therapeutic decision-making [19,27].

^18^F-luorodeoxyglucose (FDG) PET, most commonly performed as PET/CT, is the most extensively studied nuclear imaging technique in CP. FDG uptake reflects increased glucose metabolism by activated inflammatory cells, including macrophages and lymphocytes, which are abundant in active pericardial inflammation. In patients with suspected transient or inflammatory constrictive pericarditis, diffuse or focal circumferential FDG uptake along the pericardium supports an inflammatory phenotype and identifies individuals who may respond favorably to anti-inflammatory therapy rather than immediate surgical pericardiectomy [27,28]. Conversely, the absence of significant FDG uptake suggests a chronic, “burnt-out” constriction dominated by fibrosis or calcification, in which medical therapy is unlikely to reverse constrictive physiology.

Several studies have demonstrated the prognostic and therapeutic implications of FDG-PET in CP. Chang et al. showed that increased pericardial FDG uptake predicts reversibility of constrictive physiology and response to corticosteroid therapy, supporting the concept of imaging-guided therapy in pericardial disease [28]. These findings have been incorporated into contemporary multimodality imaging frameworks, which emphasize PET as a complementary tool when CMR findings are equivocal or when there is discordance between clinical presentation and structural imaging [19].

PET/CT offers the advantage of combining metabolic information with high-resolution anatomical localization provided by CT. This hybrid approach allows precise co-registration of FDG uptake with pericardial thickening, calcifications, and extracardiac findings, facilitating etiological assessment and differential diagnosis. Moreover, whole-body PET/CT enables detection of systemic inflammatory, infectious, or neoplastic processes—such as tuberculosis, malignancy, or systemic inflammatory disease—that may underlie pericardial involvement [25,27]. However, FDG-PET is limited by physiological myocardial glucose uptake, which may obscure pericardial signal despite dietary preparation protocols, and by limited specificity, as increased uptake can also be observed in neoplastic or infectious conditions.

Hybrid PET/MRI represents an emerging imaging modality that integrates the metabolic sensitivity of PET with the superior tissue characterization and functional assessment of CMR within a single examination. Recent evidence suggests that PET/MRI may be particularly valuable in inflammatory cardiac diseases, including pericarditis and myopericarditis, by allowing simultaneous assessment of pericardial inflammation (FDG uptake), edema, and fibrosis (LGE) [29]. In the context of CP, PET/MRI enables precise spatial correlation between metabolic activity and pericardial LGE, improving diagnostic confidence in cases with borderline or discordant findings on standalone imaging modalities. Furthermore, PET/MRI reduces radiation exposure compared with PET/CT, an important consideration in younger patients and those requiring serial imaging for treatment monitoring [29].

Beyond FDG, novel PET tracers are gaining interest for more specific characterization of pericardial pathology. Fibroblast activation protein inhibitors (FAPI), such as ^68^Ga-FAPI-04, target activated fibroblasts involved in tissue remodeling and fibrosis. Preliminary studies comparing ^68^Ga-FAPI PET/CT with ^18^F-FDG PET/CT suggest that FAPI tracers may offer improved target-to-background contrast and reduced myocardial interference, particularly in chronic fibro-inflammatory conditions [23,30]. In CP, FAPI uptake may reflect active fibrotic remodeling rather than pure inflammation, potentially allowing more refined phenotyping along the spectrum from inflammatory to fibrotic constriction. Although these findings are promising, clinical experience remains limited, and standardized interpretation criteria are not yet established.

Despite its strengths, nuclear imaging has important limitations in CP evaluation. PET lacks the temporal resolution required to directly assess hemodynamic features of constrictive physiology, such as ventricular interdependence or respiratory variation, and therefore cannot replace echocardiography or CMR for functional assessment. Additionally, tracer uptake patterns must be interpreted within the clinical and imaging context to avoid misclassification, particularly in patients with prior cardiac surgery or radiation therapy, where inflammatory and fibrotic processes may coexist [19,25].

## 6. Invasive Hemodynamic Assessment in Constrictive Pericarditis

Despite advances in non-invasive imaging, invasive hemodynamic assessment remains essential when imaging findings are equivocal or when differentiation from restrictive cardiomyopathy is uncertain [15,25]. Simultaneous right and left heart catheterization directly demonstrates ventricular interdependence, the hallmark of constrictive physiology [15]. Characteristic findings include discordant respiratory variation in left and right ventricular systolic pressures, elevation and near equalization of diastolic pressures across cardiac chambers, and the dip-and-plateau configuration reflecting rapid early filling followed by abrupt cessation [15]. Catheterization findings should be interpreted in conjunction with imaging markers of inflammation [19,31]. When hemodynamics confirm constriction and imaging demonstrates active inflammation, a trial of anti-inflammatory therapy is reasonable [31]. Conversely, concordant hemodynamic constriction in the absence of inflammatory imaging markers favors chronic fibrotic disease and supports early referral for pericardiectomy [19,25].

## 7. Role of Pericardial Fluid Analysis and Tissue Biopsy in Differential Diagnosis

Pericardial fluid analysis and pericardial or epicardial biopsy play a complementary role in the differential diagnosis of constrictive pericarditis, particularly when imaging findings are inconclusive or when a specific etiology must be identified to guide targeted therapy. Analysis of pericardial fluid obtained by pericardiocentesis may provide important diagnostic clues in cases of suspected infectious, malignant, or inflammatory disease, including tuberculosis, purulent pericarditis, or neoplastic involvement, which remain significant causes of constrictive physiology in selected populations [8,23,25]. Cytological examination, microbiological testing, and biochemical analysis can help distinguish exudative from transudative effusions and support etiological classification.

Histological evaluation of pericardial tissue obtained via surgical or percutaneous biopsy may be required when noninvasive assessment fails to establish a diagnosis. Biopsy can reveal specific pathological processes such as granulomatous inflammation, malignancy, radiation-induced fibrosis, or systemic inflammatory disease, thereby influencing therapeutic decisions and prognosis [5,23]. Epicardial involvement, which may contribute to persistent constriction even after pericardial drainage, can also be identified histologically in selected cases. However, both fluid analysis and biopsy are invasive procedures and are generally reserved for patients with atypical presentations, suspected secondary causes, or lack of response to standard therapy. In most cases, multimodality imaging remains the primary diagnostic approach, with invasive sampling serving as an adjunct for etiological clarification when clinically indicated [19,25].

## 8. Multimodality Imaging Approach to the Diagnosis of Constrictive Pericarditis

The diagnostic evaluation of CP increasingly relies on a multimodality imaging strategy that integrates anatomical, physiological, and biological information into a unified interpretation of pericardial disease. CP encompasses a broad spectrum of phenotypes—from chronic fibrotic constriction to reversible inflammatory forms such as transient constrictive pericarditis and ECP requiring imaging tools that assess multiple dimensions of disease expression. Accordingly, contemporary frameworks emphasize the complementary roles of echocardiography, CMR, CT, and, when appropriate, nuclear imaging such as ^18^F-FDG PET [19,20].

Echocardiography remains the initial modality due to its availability and its ability to capture the hemodynamic hallmarks of constriction in real time. Findings such as respirophasic septal shift, annulus reversus/paradoxus, and expiratory hepatic vein diastolic flow reversal ≥ 0.8 reflect ventricular interdependence and closely parallel discordant pressure patterns observed during catheterization. When these features align with clinical suspicion, echocardiography alone may suffice for diagnosis [19]. However, echocardiography offers limited characterization of pericardial thickness, inflammation, and coexistent myocardial pathology—features crucial for prognostication and therapeutic decision-making.

CMR represents the next step when echocardiography is inconclusive or when tissue characterization is required. It provides a comprehensive assessment of pericardial morphology, ventricular coupling, and inflammatory activity. Free-breathing cine imaging accurately confirms septal bounce and enhanced ventricular interdependence, while T2-weighted sequences and LGE uniquely identify pericardial edema, active inflammation, and fibrosis. These markers directly inform management: patients with an inflammatory phenotype—demonstrating edema, LGE, and elevated inflammatory indices—respond favorably to anti-inflammatory therapy, whereas those with thickened, non-enhancing fibrotic pericardium generally require surgical pericardiectomy [31].

CT complements CMR by offering superior spatial resolution for evaluating pericardial thickness and calcification. Although calcification alone is neither diagnostic nor prognostic [32], CT is essential for preoperative planning and in cases with complex postsurgical anatomy.

Nuclear imaging, particularly 18F-FDG PET, provides metabolic information that complements anatomical and functional imaging. Increased pericardial FDG uptake indicates active inflammation in acute, recurrent, and ECP [33]. PET is especially useful when CMR is contraindicated or equivocal and increasingly guides immunomodulatory therapy in the era of IL-1 inhibitors. Hybrid PET/MR platforms further enhance characterization by combining metabolic and tissue-level data.

**Figure 4:**
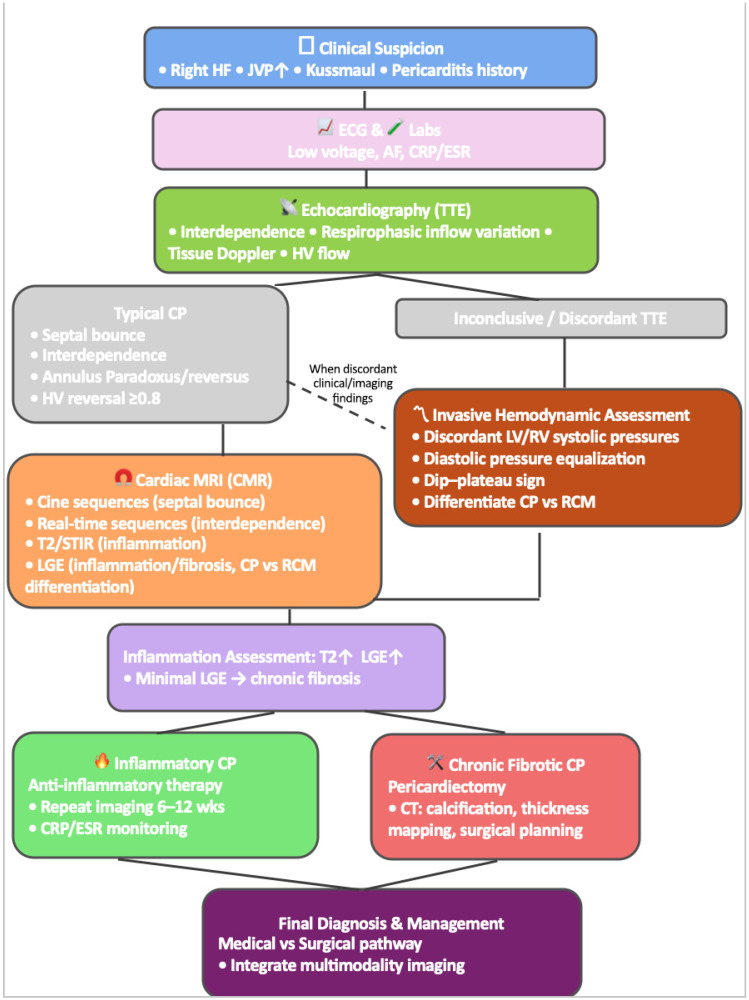
Diagnostic Flow chart in CP.

The strength of a multimodality approach lies in its ability to synthesize these complementary findings into a coherent diagnostic pathway (Table 1, Figure 4). When echocardiography clearly demonstrates constrictive physiology, further testing may be unnecessary. In cases of ambiguous or discordant findings, CMR is the preferred modality to confirm constriction and assess reversibility [19]. CT is incorporated when calcification or surgical planning is relevant, while PET is reserved for detailed inflammatory assessment. Multimodality imaging also remains fundamental for differentiating CP from RCM—an essential distinction because therapeutic strategies diverge substantially.

### 8.1. Multimodality Imaging for Differentiating Constrictive Pericarditis from Restrictive Cardiomyopathy

A direct comparison between CP and RCM requires integration of findings across imaging modalities, as each technique interrogates different aspects of pathophysiology. Echocardiography primarily evaluates dynamic hemodynamics and ventricular interdependence, demonstrating characteristic features of CP such as septal bounce, marked respiratory variation in transvalvular flows, preserved or increased medial e′ velocity, and annulus reversus or paradoxus, whereas RCM typically shows reduced e′ velocities, minimal respiratory variation, and severe biatrial enlargement reflecting intrinsic myocardial stiffness [11,17,25]. However, echocardiography alone may be inconclusive in localized constriction or in advanced myocardial disease.

Cross-sectional imaging provides complementary structural and tissue-level information. CMR distinguishes CP from RCM by directly visualizing pericardial pathology and myocardial involvement: pericardial thickening, septal shift with respiration, and pericardial late gadolinium enhancement favor CP, while myocardial infiltration or fibrosis patterns—such as subendocardial or diffuse enhancement—support RCM [19,22,25]. Importantly, CMR can identify active inflammation, indicating potentially reversible constriction, a feature not present in primary myocardial restrictive diseases [20,31].

Cardiac CT further refines anatomical assessment, particularly through its superior ability to detect pericardial calcification and delineate the distribution of constriction. Extensive calcification strongly favors chronic CP and is not a feature of RCM, whereas normal pericardial morphology in the presence of restrictive physiology should prompt consideration of myocardial disease [23,25]. CT also aids in excluding alternative extracardiac causes of right-sided heart failure that may mimic either condition [24].

Nuclear imaging contributes biological information by identifying metabolically active inflammation. Increased pericardial FDG uptake supports an inflammatory CP phenotype with potential reversibility, whereas the absence of uptake is consistent with chronic fibrotic constriction; RCM, in contrast, typically demonstrates myocardial rather than pericardial metabolic abnormalities depending on the underlying infiltrative process [27,28]. Because PET does not assess ventricular interdependence directly, its findings must be interpreted alongside structural and functional imaging.

Ultimately, no single modality is sufficient to reliably distinguish CP from RCM in all cases. A stepwise multimodality approach integrating echocardiographic hemodynamics, CMR tissue characterization, CT anatomical detail, and PET inflammatory assessment provides the most robust diagnostic framework, reduces misclassification, and guides appropriate therapeutic decision-making, including the identification of patients who may benefit from anti-inflammatory therapy versus surgical pericardiectomy [19,25,26].

### 8.2. Multimodality Imaging in Post-Treatment Follow-Up of Constrictive Pericarditis

Imaging also plays a crucial role during follow-up after both medical therapy and surgical pericardiectomy, as clinical improvement does not always parallel normalization of pericardial physiology. After anti-inflammatory treatment for transient or subacute CP, echocardiography is typically the first-line modality for serial assessment of ventricular interdependence, respiratory variation in transvalvular flows, and inferior vena cava dynamics, allowing noninvasive monitoring of hemodynamic recovery [19,25]. Resolution or marked reduction in septal bounce and respiratory Doppler abnormalities supports reversibility, whereas persistence of constrictive features suggests incomplete response or progression toward chronic disease.

CMR provides the most comprehensive evaluation of treatment response by integrating functional assessment with tissue characterization. Reduction in pericardial edema on T2-weighted imaging and decreasing late gadolinium enhancement over time correlate with resolution of inflammation and improvement in constrictive physiology, while persistent enhancement indicates ongoing inflammatory activity or evolving fibrosis [6,22,31]. Serial CMR is therefore particularly valuable when clinical symptoms and echocardiographic findings are discordant or when therapeutic escalation versus surgical referral is being considered [19,20].

Cardiac CT is not routinely used for longitudinal inflammatory monitoring but may be helpful in selected cases to evaluate progression of calcification, assess residual pericardial thickening, or define postoperative anatomy after pericardiectomy, especially when CMR is contraindicated [23,25]. Nuclear imaging with FDG-PET can further identify residual or recurrent inflammatory activity, which may guide continuation or intensification of anti-inflammatory therapy in patients with persistent symptoms despite equivocal structural imaging findings [27,28].

Following surgical pericardiectomy, echocardiography remains the primary modality for postoperative assessment, allowing evaluation of ventricular filling dynamics, residual constrictive physiology, and potential complications such as right ventricular dysfunction or persistently elevated filling pressures [5,25]. Cross-sectional imaging may be required when symptoms persist, to distinguish incomplete pericardial resection from myocardial disease or postoperative scarring. Overall, a tailored multimodality strategy—guided by clinical status, suspected disease activity, and prior imaging findings—provides the most effective approach for post-treatment surveillance and long-term management of CP [19,26].

### 8.3. Preoperative Imaging and Surgical Prognosis

Preoperative multimodality imaging also provides important prognostic information that may influence surgical risk stratification and expected outcomes after pericardiectomy in patients with chronic CP. Extensive pericardial calcification, diffuse circumferential involvement, and dense adhesions identified on CT are associated with increased technical complexity, incomplete decortication, and higher perioperative morbidity [23,25,32]. Similarly, CMR findings suggestive of myocardial atrophy, fibrosis, or concomitant myocardial disease may indicate reduced ventricular compliance independent of pericardial constraint and have been linked to less favorable functional recovery following surgery [19,25]. Conversely, imaging evidence of active inflammation without advanced calcification may identify patients earlier in the disease course who are more likely to benefit from surgical intervention with improved postoperative hemodynamics.

Assessment of disease extent and involvement of critical structures—such as the atrioventricular grooves, diaphragmatic surface, and regions adjacent to the phrenic nerves or coronary arteries—is particularly relevant for operative planning and prediction of residual constriction [23]. Furthermore, multimodality imaging can help distinguish patients with predominant pericardial pathology from those with mixed myocardial–pericardial disease, a distinction that strongly influences long-term outcomes after pericardiectomy [5,26]. In addition, comprehensive preoperative imaging is essential for detecting concomitant coronary artery disease or clinically significant valvular abnormalities, which may substantially influence surgical planning, perioperative risk assessment, and the need for concomitant coronary or valvular procedures at the time of pericardiectomy [5,19,23]. Thus, comprehensive preoperative imaging not only confirms the diagnosis but also provides essential information for patient selection, surgical planning, and prognostic counseling in chronic constrictive pericarditis.

## 9. Current and Future Applications of Artificial Intelligence in Multimodality Imaging for CP

Artificial intelligence (AI) is increasingly being integrated into cardiovascular imaging workflows and holds particular promise in the evaluation of CP, a rare and diagnostically challenging condition that relies on the interpretation of complex and subtle imaging features across multiple modalities. The diagnosis of CP requires recognition of characteristic but often nuanced morphological, functional, and hemodynamic patterns, a process that is highly operator-dependent and subject to interobserver variability. In this context, AI-based approaches may enhance diagnostic accuracy, improve reproducibility, and support clinical decision-making by enabling data-driven pattern recognition beyond conventional single-parameter assessment [27,34].

Most early applications of AI in CP have focused on echocardiography, reflecting its central role as first-line imaging and the richness of functional information it provides. A pioneering machine-learning study using speckle-tracking–derived deformation parameters demonstrated the feasibility of differentiating CP from RCM by integrating multiple echocardiographic features into a unified diagnostic framework [35]. More recently, deep learning models based on convolutional neural networks have enabled end-to-end analysis of raw transthoracic echocardiographic images. In a large single-center study, a deep learning model trained on standard apical four-chamber views accurately differentiated CP from cardiac amyloidosis, achieving excellent diagnostic performance with an area under the curve of 0.97 and demonstrating robust external validation [36]. Importantly, explainability analyses revealed that model attention was focused on clinically relevant regions such as the interventricular septum, supporting biological plausibility and interpretability.

Beyond echocardiography, AI is increasingly applied to cross-sectional imaging. In cardiac CT, deep learning–based segmentation and functional analysis algorithms have shown high accuracy in quantifying ventricular volumes, myocardial strain, and wall thickening from four-dimensional datasets, even at substantially reduced radiation doses [37]. Although not specifically developed for CP, these techniques provide a technological foundation for future AI-assisted functional CT evaluation of constrictive physiology, potentially improving assessment of ventricular coupling and septal dynamics while enhancing dose efficiency. Similarly, in nuclear imaging, AI-based tools are being explored for automated segmentation, reconstruction, and quantitative analysis of inflammatory activity on PET/CT and PET/MRI, with potential applications in identifying active pericardial inflammation and monitoring response to anti-inflammatory therapy [27].

A major future direction lies in multimodal AI frameworks capable of integrating echocardiographic, CT, CMR, and PET data with clinical and laboratory information. Such approaches are particularly attractive in CP, where disease phenotype, chronicity, and reversibility vary widely and directly influence therapeutic strategy [19]. Despite these advances, current limitations—including small datasets, lack of standardized protocols, and limited external validation—underscore that AI should presently be viewed as a complementary decision-support tool rather than a replacement for expert clinical interpretation. As multimodality imaging datasets expand and methodological rigor improves, AI is poised to play an increasingly important role in precision imaging and personalized management of constrictive pericarditis.

## 10. Conclusions

Constrictive pericarditis remains a diagnostically challenging and clinically heterogeneous condition, in which timely recognition and accurate phenotyping are essential to optimize patient outcomes. No single imaging modality is sufficient to fully characterize the complex interplay between pericardial anatomy, ventricular mechanics, and inflammatory activity that defines the spectrum of constrictive disease. A multimodality imaging approach, integrating echocardiography, CMR, cardiac CT, and nuclear imaging, provides complementary and synergistic information that enhances diagnostic confidence and guides personalized management strategies.

Echocardiography remains the cornerstone for identifying constrictive physiology through real-time hemodynamic assessment, while CMR plays a central role in tissue characterization and differentiation between inflammatory and fibrotic phenotypes. Cardiac CT offers unparalleled evaluation of pericardial calcification and surgical anatomy, and nuclear imaging uniquely identifies metabolically active inflammation, supporting imaging-guided therapy. Emerging applications of artificial intelligence further promise to refine disease phenotyping and improve diagnostic reproducibility. Together, these advances underscore the pivotal role of multimodality imaging in enabling precision diagnosis, appropriate therapeutic selection, and improved outcomes in patients with constrictive pericarditis.

## Figures and Tables

**Table 1:** Comparative Advantages and Limitations of Multimodality Imaging in the Diagnosis of Constrictive Pericarditis.

Imaging Modality	Key Advantages	Main Limitations	Key Diagnostic Criteria (Practical Thresholds)
**Echocardiography**	First-line, non-invasive and widely available technique; real-time assessment of hemodynamics and respiratory variation; key role in identifying ventricular interdependence and differentiating CP from RCM; allows evaluation of myocardial strain and hepatic vein flow patterns	Limited ability to directly assess pericardial anatomy; operator- and image-quality dependent; hallmark findings may be absent in localized constriction or masked by coexisting conditions; inability to identify active pericardial inflammation or fibrosis	Septal bounce; Mitral inflow respiratory variation ≥ 25%; Tricuspid inflow variation ≥ 40%; Hepatic vein expiratory diastolic reversal ratio ≥ 0.8; medial e′ ≥ 8 cm/s; annulus reversus (medial e′ > lateral e′); annulus paradoxus (low E/e′ despite high filling pressure); strain reversus ratio < 0.96
**Cardiac Magnetic Resonance (CMR)**	Comprehensive assessment of pericardial anatomy, ventricular interaction, and myocardial–pericardial coupling; superior tissue characterization with edema detection and late gadolinium enhancement; differentiation between inflammatory and fibrotic phenotypes; key tool for therapy guidance and follow-up	Limited availability; contraindications in patients with non-compatible devices or severe claustrophobia; longer acquisition times; reduced sensitivity for calcification	Real-time septal shift with respiration (ventricular interdependence); pericardial thickness > 4 mm (supportive); T2/STIR hyperintensity = active inflammation; pericardial LGE = inflammatory phenotype; absent LGE = fibrotic constriction
**Cardiac Computed Tomography (CCT)**	Reference standard for detection and characterization of pericardial calcifications; high spatial resolution for pericardial anatomy; whole-heart and extracardiac assessment; essential for preoperative planning; emerging capability for functional and inflammatory assessment with advanced techniques	Exposure to ionizing radiation; limited soft tissue characterization compared with CMR; requires iodinated contrast; functional assessment inferior to echo and CMR; limited role in longitudinal monitoring of inflammation	Pericardial thickening (>4 mm) or calcification; focal vs. circumferential constriction mapping for surgical planning; iodine mapping or delayed enhancement suggesting inflammation (emerging)
**Nuclear Imaging (PET/CT–PET/MRI)**	Direct visualization and quantification of metabolically active pericardial inflammation; identification of reversible inflammatory constriction; guidance of imaging-based therapeutic decisions; whole-body evaluation for etiologic diagnosis; PET/MRI enables combined metabolic and tissue characterization	Limited availability and higher cost; radiation exposure (lower with PET/MRI); limited specificity of tracer uptake; physiological myocardial uptake may interfere with interpretation; no direct assessment of hemodynamic constrictive physiology	Focal pericardial FDG uptake → active inflammatory CP; absence of uptake → chronic fibrotic disease; diffuse myocardial uptake → inadequate preparation

## Data Availability

No new data were created or analyzed in this study. Data sharing is not applicable to this article.

## Abbreviations

The following abbreviations are used in this manuscript:

CP	Constrictive pericarditis
RCM	restrictive cardiomyopathy
CT	Computed Tomography
CMR	Cardiac magnetic resonance
LGE	late gadolinium enhancement
PET	positron emission tomography
ECP	Effusive-constrictive pericarditis
LV	Left ventricular
FDG	fluorodeoxyglucose
FAPI	Fibroblast activation protein inhibitors
AI	Artificial intelligence
